# Bandpass Shape of Distortion-Product Otoacoustic Emission Ratio Functions Reflects Cochlear Frequency Tuning in Normal-Hearing Mice

**DOI:** 10.1007/s10162-023-00892-4

**Published:** 2023-04-18

**Authors:** James B. Dewey, Christopher A. Shera

**Affiliations:** 1 Caruso Department of Otolaryngology - Head and Neck Surgery, University of Southern California, Los Angeles, 90033 CA USA; 2 Department of Physics and Astronomy, University of Southern California, Los Angeles, CA 90089 USA

**Keywords:** Frequency selectivity, Distortion-product otoacoustic emission, Cochlea, Outer hair cell, Optical coherence tomography

## Abstract

The frequency selectivity of the mammalian auditory system is critical for discriminating complex sounds like speech. This selectivity derives from the sharp tuning of the cochlea’s mechanical response to sound, which is largely attributed to the amplification of cochlear vibrations by outer hair cells (OHCs). Due to its nonlinearity, the amplification process also leads to the generation of distortion products (DPs), some of which propagate out to the ear canal as DP otoacoustic emissions (DPOAEs). However, the insight that these signals provide about the tuned micro- and macro-mechanics underlying their generation remains unclear. Using optical coherence tomography to measure cochlear vibrations in mice, we show that the cochlea’s frequency tuning is reflected in the bandpass shape that is observed in DPOAE amplitudes when the ratio of the two evoking stimulus frequencies is varied (here termed DPOAE “ratio functions”). The tuning sharpness of DPOAE ratio functions and cochlear vibrations co-varied with stimulus level, with a similar quantitative agreement in tuning sharpness observed for both apical and mid-cochlear locations. Measurement of intracochlear DPs revealed that the tuning of the DPOAE ratio functions was not caused by mechanisms that shape DPs locally near where they are generated. Instead, simple model simulations indicate that the bandpass shape is due to a more global wave interference phenomenon. It appears that the filtering of DPOAEs by wave interactions over an extended spatial region allows them to provide a window onto the frequency tuning of single cochlear locations.

## Introduction

In mammals, the ability to discriminate and segregate sounds with similar frequency content depends on an active amplification process mediated by the cochlear outer hair cells (OHCs) [1]. While passive gradients in the stiffness of the basilar membrane (BM) underlie the cochlea’s fundamental frequency-to-place mapping, such that low-frequency sounds elicit waves on the BM that peak at the cochlear apex, and high-frequency sounds elicit waves that peak toward the base [2], the frequency tuning of each location is dramatically enhanced by the OHCs. OHCs first detect vibrations of the underlying BM via deflection of their mechanotransducing stereociliary bundles and then generate force in response [3, 4]. These forces amplify traveling waves in a frequency-selective manner [1, 5, 6], thus increasing and sharply tuning the mechanical input to the inner hair cells (IHCs), the cochlea’s primary afferent receptors communicating with the auditory nerve.

While responsible for the cochlea’s remarkable frequency selectivity, the amplification process is also highly nonlinear, resulting in the generation of distortion products (DPs) at frequencies not present in the acoustic stimulus. The nonlinearity is primarily attributed to OHC mechanotransduction, which introduces DPs into the electrical potentials that drive OHC force generation, thus initiating waves at DP frequencies [7]. Some of these waves travel to the stapes and are transmitted to the ear canal where they can be recorded as distortion-product otoacoustic emissions (DPOAEs) [8]. Typically elicited by two tones at frequencies *f*_1_ and *f*_2_ (*f*_2_ > *f*_1_) and measured at related frequencies such as 2*f*_1_ – *f*_2_, DPOAEs provide a noninvasive window onto cochlear mechanics. However, due to complexities in how these signals are generated and propagate, it remains uncertain whether DPOAEs provide any precise information about the nonlinear, frequency-selective processes that generate them. To address this, the present report examines a phenomenon observed in DPOAE recordings that has long been suggested to relate to cochlear frequency tuning — namely, the bandpass shape observed in 2*f*_1_ – *f*_2_ DPOAE amplitudes as the ratio of the two stimulus frequencies is varied from large to small [9–12].

Originally attributed to a resonance of the tectorial membrane (TM) overlying the OHCs [13–15], the bandpass shape in DPOAE amplitude vs. *f*_2_/*f*_1_ ratio functions (referred to here as “ratio functions”) has also been proposed to be due to suppression [16], the form of the underlying nonlinearity [17], and interference between DP waves generated at different locations [18–21]. These mechanisms all theoretically depend on or involve the sharpness of cochlear tuning, suggesting a relationship between the tuning of cochlear vibrations and DPOAE ratio functions. Though the tuning of DPOAE ratio functions is only modestly correlated with psychophysical measures of tuning in humans [22, 23], the latter are likely influenced by central properties, and it is unclear how they relate to cochlear tuning, specifically. Modeling of human DPOAE generation suggests that cochlear tuning can be predicted from DPOAE ratio functions [24]; however, the necessary assumptions regarding human cochlear mechanics remain speculative.

Here, we explicitly tested this relationship in mice by using optical coherence tomography to measure cochlear vibrations, and then comparing the tuning of these responses with the tuning of DPOAE ratio functions measured in the same ears. Additionally, we used intracochlear DP measurements and a simple model to test whether DPOAE ratio functions are tuned by local mechanisms that shape DPs where they are generated, or by a more global mechanism such as wave interference.

## Methods

### Mouse Preparation

DPOAEs and cochlear vibrations were measured from adult (4–7 week old) wild-type CBA/CaJ mice of either sex (*n* = 29, 15 female). Mice were bred and housed on-site at the University of Southern California, and all procedures were approved by the local Institutional Animal Care and Use Committee.

Mice were anesthetized (80 mg/kg ketamine, 10 mg/kg xylazine) and placed on a heating pad to maintain body temperature at ~ 38 °C, with additional anesthesia administered to ensure areflexia for the duration of the experiment. After fixing the skull to a head-holder with dental cement, the left bulla was accessed using a ventrolateral surgical approach. The bone below the tympanic annulus was then removed to widely expose the middle ear space and otic capsule. Resection of the pinna and ear canal facilitated placement of the tip of an acoustic probe (ER10-X; Etymotic Research, Elk Grove, IL) within a few mm of the tympanic membrane, with the probe tip coupled to the residual ear canal via plastic tubing. The tubing was glued in place to create a stable and closed acoustic field. After all desired measurements were completed, mice were euthanized by anesthetic overdose. Measurements were often repeated postmortem to ensure the absence of any artifactual distortion in the in vivo responses. Postmortem vibrations of the ossicular chain were also measured in a subset of mice in order to quantify delays associated with middle-ear transmission.

### Optical Coherence Tomography

Cochlear vibrations were measured in response to single- and two-tone stimuli using a custom-built optical coherence tomography (OCT) system that has previously been described in detail [5, 25–27]. Briefly, the system employs an akinetic swept laser with a 1310-nm center wavelength, 95-nm bandwidth, and 100-kHz sweep rate (Insight Photonic Solutions, Inc., Lafayette, CO). Light reflected from the sample was combined with the light from a reference mirror in order to generate interferograms, which were digitized at 12-bit resolution and 400 MS/s (AlazarTech ATS9373 card; Alazar Technologies Inc., Pointe-Claire, QC, Canada). Custom software (written in Python, CUDA, and C++) was used in conjunction with National Instruments hardware (Austin, TX) to generate stimulus waveforms, control the mirror used to scan the laser across the sample, and process the interferometric signals. The axial and lateral imaging resolutions of the system were ~ 12.5 and ~ 9.8 μm, respectively, and the displacement measurement noise floor was typically ~ 0.01 to 0.1 nm depending on the amount of time-domain averaging used and the reflectivity of the measurement location.

**Fig. 1:**
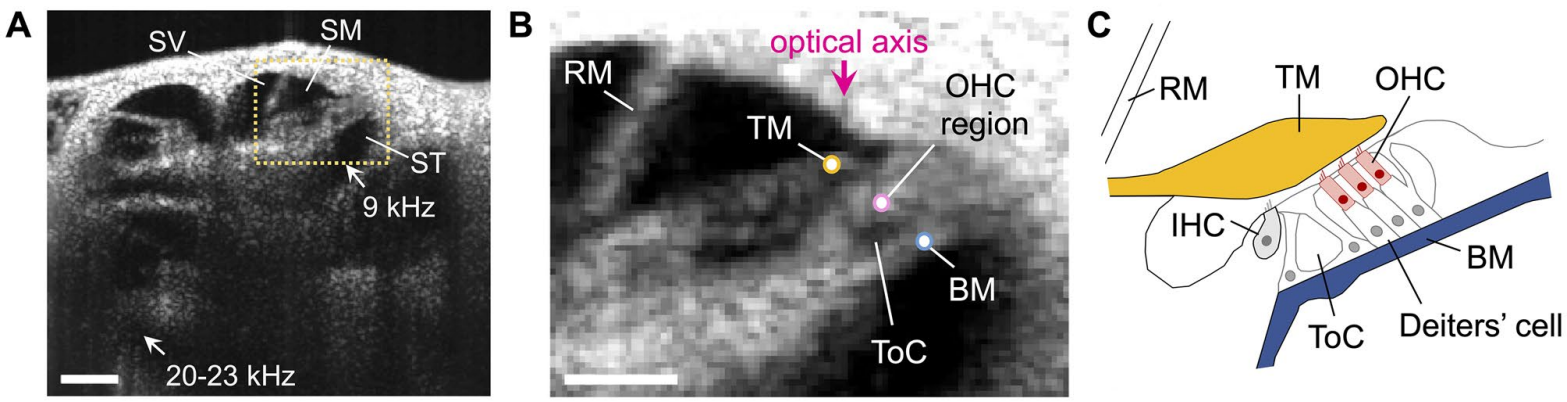
OCT imaging of the mouse cochlea. **A** Cross-sectional OCT image of the live mouse cochlea. The three fluid-filled scalae are labeled (SV = scala vestibuli, SM = scala media, ST = scala tympani). Highlighted is the apical region tuned to 9 kHz, where vibrations were typically measured. Also indicated is the middle-turn region tuned to 20–23 kHz, where limited additional measurements were made. Scale bar = 200 μm. **B** Magnified cross-section of the 9 kHz region highlighted in (**A**). Indicated are the approximate points on the BM, OHC region, and TM from which vibrations were measured. Vibration measurements captured motions in line with the optical axis. Also indicated are the tunnel of Corti (ToC) and Reissner’s membrane (RM). Scale bar = 100 μm. **C** Schematic showing the relevant anatomy

After opening the bulla, the laser was scanned across the cochlear bone to obtain cross-sectional images (Fig. 1A). The mouse’s head was adjusted so that the BM in the apical turn was oriented roughly orthogonal to path of the laser, with the angle between the BM surface and the beam path being at least 60°. Only displacements in line with the beam path were measured, such that the recordings were likely dominated by the transverse motion of the structures. However, radial and/or longitudinal motions may also have contributed to the measured signal.

We primarily measured vibrations from an apical cochlear region tuned to a characteristic frequency (CF) of 9 kHz, where displacements were measured from the BM, OHC region, and TM (Fig. 1B, C). Positions of various measurement points were inferred from the known anatomy [28]. Measurements were obtained from the BM near its midpoint, where vibrations are maximal [25], while OHC region measurements were obtained near the junction between the OHCs and their underlying supporting cells (the Deiters’ cells), which connect the OHCs to the BM. TM measurements were obtained from a point above the apical ends of the OHCs, roughly halfway between the TM’s lower and upper surfaces. Since it was not always possible to clearly visualize the TM, measurements may have been closer to the lower or upper surface in certain preparations. To ensure a high signal-to-noise ratio, measurements were always obtained from highly reflective points (i.e., bright pixels in the cross-sectional images).

In six mice, we obtained vibrations from mid-cochlear regions with CFs of 20–23 kHz, which we will refer to as “middle-turn” locations. Since the light reflected from the middle turn was weak, it was difficult to discern any individual structures or features, with the BM and organ of Corti appearing as a grainy, partially obscured mass. Nevertheless, interpretable vibration data were obtained from points at the bottom or top of the partition, and the responses exhibited nonlinear characteristics similar to those observed in the apical turn.

### Single- and Two-Tone Stimulus Paradigms

Responses to single tones were used to quantify cochlear frequency tuning, while two-tone stimuli were used to elicit intracochlear DPs and DPOAEs. All stimuli were 102 ms in duration, with 1 ms ramps applied to the beginning and end of the stimulus waveform, and were presented once every ~ 110 ms. Stimuli were calibrated using the pressure measured by the probe microphone, after compensating for its frequency-dependent transfer function.

In apical measurements, single-tone responses were obtained at frequencies from 1 to 15 kHz in 0.5 kHz steps. We first obtained BM responses for tones presented at 30 dB sound pressure level (SPL) so as to determine the CF of the measurement site, which was defined as the frequency eliciting the maximum response. Displacements were then obtained from the BM, OHC region, and TM with stimuli presented from 10 to 90 dB SPL in 10 dB steps. In later experiments, additional measurements were obtained from 30 to 75 dB SPL in 5 dB steps, so as to cover the same range of same stimulus levels over which DPOAEs were also typically measured. In middle-turn measurements, single-tone responses were obtained from 1 to 30 kHz in 0.5 or 1 kHz steps with stimulus levels varied from 30 to 90 dB SPL in 10 dB steps. Single-tone responses were averaged over 1–16 stimulus repetitions, with 8 stimulus repetitions being used in the majority of measurements.

Two-tone responses were obtained with the higher-frequency *f*_2_ tone fixed near the CF of the measurement site and the *f*_1_ tone varied to achieve *f*_2_/*f*_1_ ratios of ~ 1.02–1.87 in 0.025 steps. In one paradigm, the levels of the *f*_1_ and *f*_2_ tones were the same and varied from 30 to 70 dB SPL in 10 dB steps or 30 to 75 dB SPL in 5 dB steps. In a second paradigm used to examine the influence of suppression, the level of the *f*_2_ tone was fixed at 60 dB SPL and the *f*_1_ tone was varied from 45 to 75 dB SPL in 5 dB steps. Responses were averaged over 8–32 stimulus repetitions.

For all measurements, a fast Fourier transform was applied to the steady-state portion of the average response in order to extract the magnitude and phase at the stimulus and DP frequencies. Stimulus frequencies were rounded to the nearest 10 Hz so that the steady-state response included an integer number of stimulus cycles. The noise floor for each response component was taken as the mean + 3 standard deviations (SDs) of the magnitudes in nearby frequency bins (± 220–320 Hz for responses at the stimulus frequencies, and ± 20–120 Hz for DPs). Response phases were referenced to the phase of the stimulus pressure measured in the ear canal. For DPs and DPOAEs at 2*f*_1_ – *f*_2_, this involved subtracting 2*φ*_1ec_ – *φ*_2ec_ from the response phase, where *φ*_1ec_ and *φ*_2ec_ were the phases of the *f*_1_ and *f*_2_ stimuli in the ear canal, respectively.

Initially, vibratory and ear-canal responses to two-tone stimuli were only obtained simultaneously. However, we found that keeping the number of stimulus repetitions to ~ 8–16 was necessary to cover all stimulus frequencies and levels without drift in the reflectivity of the measurement point over time. While avoiding such drift was required to obtain clean vibratory responses, using a low number of stimulus repetitions also resulted in a higher noise floor in the DPOAE measurements. Acoustic noise from the OCT system’s scanning mirror and electrical noise from National Instruments hardware were also sometimes problematic. Thus, in later experiments, we obtained DPOAE measurements using an RME Babyface audio interface (Audio AG, Haimhausen, Germany) and software (ARLas, provided by Dr. Shawn Goodman, https://github.com/myKungFu/ARLas) written in MATLAB (MathWorks, Natick, MA), using a 192-kHz sampling rate. Responses were averaged over 32 stimulus repetitions but were otherwise obtained using identical parameters, and the resulting DPOAEs were consistent with those obtained during the two-tone OCT measurements. The reduced noise floors in the RME audio interface recordings facilitated calculation of DPOAE tuning sharpness at lower stimulus levels.

For middle-turn measurements, the higher displacement noise floors precluded characterization of the intracochlear DPs, such that two-tone responses were only obtained from the ear canal. For these measurements, we first used OCT to measure single-tone vibratory responses, and then used the RME interface to obtain DPOAE ratio functions with *f*_2_ set near the CF of the measurement site. As the vibratory measurements from the middle turn were somewhat challenging, multiple attempts to record clean responses were made both before and after the DPOAE measurements, which sometimes involved repositioning of the mouse to improve the OCT image. Thus, the CF of the recording site which ultimately yielded the cleanest data was not always identical to the *f*_2_ frequency used in the DPOAE measurements, differing by ~ 1 kHz (~ 0.1 octave) in three of the six mice. Since cochlear tuning is expected to vary only slowly with location [29], such differences likely have little impact on the results.

As we did not wish to artificially minimize variability in cochlear sensitivity and tuning across mice, we did not first obtain auditory brainstem responses to screen for normal hearing function. Nevertheless, all preparations were considered to have essentially normal mechanical sensitivity, with displacement responses to low-level tones varying by less than 12 dB for a given measurement location. For apical measurements, BM responses to CF tones presented at 30 dB SPL ranged in magnitude from 0.9 to 2.6 nm, falling at most 2.6 dB below to 6.4 dB above the average response (1.24 nm). In middle-turn measurements from both the bottom and top of the organ of Corti, CF responses to 30 dB SPL tones ranged in magnitude from 0.3 to 1.12 nm, falling at most 4.8 dB below to 6.7 dB above the average response (0.52 nm). When obtained using the optimal *f*_2_/*f*_1_ ratio for a given *f*_2_ frequency, DPOAE amplitudes always exceeded -5 dB SPL for equal-level stimuli presented at 40 dB SPL or higher. For a given *f*_2_ frequency and stimulus level, DPOAE amplitudes all fell within a 10-dB range.

### Quantifying Tuning Sharpness

We quantified the tuning of vibratory responses to single tones by dividing the frequency eliciting the peak displacement response by the bandwidth 10 dB below the peak, yielding the *Q*_10dB_. For DPOAE measurements, we calculated *Q*_10dB_ in the same manner, with DPOAE amplitudes plotted and analyzed with respect to the DP frequency. To verify that the results held across different metrics of tuning, we also calculated the equivalent rectangular bandwidth (ERB) of the responses by normalizing the amplitudes (in linear units) to their maximum, squaring the normalized amplitudes to be proportional to power, and then computing the area under the curve. Dividing the response’s peak frequency by the ERB yielded the *Q*_ERB_. When multiple measurements of either DPOAE ratio functions or vibratory responses were obtained in a single preparation, *Q*_10dB_ or *Q*_ERB_ was calculated for each individual measurement and then averaged across all measurements to obtain the final value (*n* = 1–5 across preparations).

Unlike in recent human studies [23, 24], we quantified the tuning of DPOAE ratio functions without first attempting to the reduce the influence of so-called "reflection" components in the responses, which arise from backscattering of DP waves as they propagate apically to the place tuned to the DP frequency [30]. These reflections can interfere with components originating from near the place tuned to *f*_2_ to produce peaks and valleys in the measured responses, potentially complicating tuning estimates. While signal-processing techniques can help to separate and attenuate the reflection component based on its distinct phase-vs.-frequency behavior [24], we did not apply these techniques in the present study, as the delays of emissions arising from reflection are very short (< 1 ms) in mice [31], making it difficult to cleanly separate the reflection component. Furthermore, such component separation appeared unnecessary, as DPOAE amplitudes typically varied smoothly with frequency, indicating that any reflection components were negligible. This is consistent with previous studies in other rodents, which have also found that reflection components are small [32]. Though ratio functions obtained with high *f*_2_ frequencies sometimes exhibited stronger amplitude fluctuations, these typically occurred at DP frequencies distant from the dominant peak of the functions and so were unlikely to influence tuning estimates. Thus, for simplicity, we quantified tuning using the raw ratio functions.

### Modeling Wave Interference

We implemented a simple model in MATLAB to determine the influence of wave interference on DPOAEs. Using the frequency-to-place map of the mouse cochlea [33], we estimated the displacements evoked by a given pair of *f*_1_ and *f*_2_ tones as a function of cochlear distance (0–5.13 mm from the base, in 0.01 mm steps) by extrapolating from average BM frequency responses (*n* = 22) at the 9-kHz location. To ensure that the BM phases only reflected delays associated with traveling-wave propagation, the average phases of postmortem middle-ear vibrations (*n* = 22) were first subtracted from the BM responses. Averaged across frequency, the mean middle-ear delay was ~ 36 μs.

To generate DPs at each cochlear location, displacements at *f*_1_ and *f*_2_ were given as inputs to a first-order Boltzmann function, which we used to approximate the nonlinear relationship between OHC stereociliary bundle deflection and the resulting mechanotransduction current. The function is given by:

1 $$y(x) = 1/(1 + {e}^{{a}_{1}({x}_{1}\,-\,x)}),$$

where *y* is the current, *x* is the instantaneous bundle position, *a*_1_ sets the slope of the function (here, 0.28 nm^−1^), and *x*_1_ is the position of maximum slope (here, 2.6 nm). Since the changes in membrane potential that drive OHC force generation are low-pass filtered by the membrane’s electrical properties [34–36], the Boltzmann’s output was low-pass filtered using a corner frequency 2.36 octaves below the local CF. The Boltzmann and low-pass filter parameters were derived in a previous analysis of vibratory nonlinearities in the 9-kHz location [25]. While this analysis focused on fitting the parameters to replicate the level-dependent growth of harmonic and tonic distortions in single-tone responses of the OHC region, these same parameters have recently been shown to also approximate the growth of the 2*f*_1_ – *f*_2_ DP [37].

The magnitude and phase of the low-pass filtered output at 2*f*_1_ – *f*_2_ were taken to be those of the locally generated DP. To estimate the DP arriving at the stapes, we assumed that each locally generated DP produced a reverse-propagating wave coupled to BM motion. Thus, DP magnitudes at each location were scaled by the BM displacement that would be driven by a low-level (40 dB SPL) tone at the DP frequency. Likewise, the phase of each DP was shifted to account for the phase delay due to reverse propagation, which we assumed to be the local phase of a forward-traveling BM wave at the DP frequency. These adjustments are admittedly crude and ignore the physics of how OHC generated forces couple to BM motion and produce the pressure waves that lead to DPOAEs (e.g., [38]). However, as mentioned below, exploration of the model’s output indicates that such details are unlikely to fundamentally change the predicted effects of wave interference. To estimate the total resulting DPOAE, we vectorially summed the BM-displacement-weighted DPs arriving at the stapes from all locations.

Though the assumption of reverse DP propagation primarily through “slow” traveling waves is supported by abundant experimental data and modeling efforts [39–44], an alternative hypothesis is that DPs propagate to the stapes through “fast” compression waves in the cochlear fluid [45, 46]. DPs propagating in reverse through fast waves would experience little delay, potentially leading to differences in how they interfere. In the end, however, assuming reverse DP propagation through either fast or slow waves did not qualitatively affect the model’s output, and, thus, did not alter our primary conclusions.

We also tested whether similar results were obtained when using average middle-turn vibrations (*n* = 6) to estimate DPOAE ratio functions for an *f*_2_ of 20 kHz. The phases of middle-turn vibratory responses below or above 15 kHz were corrected using average middle-ear measurements obtained in 22 or 10 mice, respectively. Responses from different locations were then expressed in octaves relative to the CF and interpolated prior to averaging. Due to the lower signal-to-noise ratio and phase irregularities at frequencies above the CF, it was unfortunately not possible to estimate traveling wave responses at locations greater than 0.32 mm apical to each response’s peak. This effectively limited the DP generation in the model to locations no more than 0.32 mm apical to the *f*_2_ place. However, we do not anticipate that this significantly affected the estimated ratio functions, as the magnitudes and phases of the modeled DPs indicated that the dominant DPOAE sources were basal to this location. Moreover, imposing a similar limitation on the model using the apical data had little effect on the estimated ratio functions.

### Experimental Design and Statistical Analyses

Statistical analyses were performed using MATLAB and SPSS (IBM Corp, Armonk, NY). Vibratory and DPOAE *Q*_10dB_ values were compared with repeated-measures ANOVA, with stimulus level and measurement location (i.e., BM, OHC region, TM, and ear canal) as within-subjects factors. Vibratory *Q*_10dB_ values for apical and middle-turn CF regions were compared using a two-way mixed ANOVA, with CF (apical vs. middle turn) as a between-subjects factor and stimulus level as a within-subjects factor. Results of repeated-measures ANOVA were adjusted for violations of sphericity using Greenhouse–Geisser corrections when applicable, and pairwise comparisons were performed using Bonferroni corrections. Pearson’s correlations were applied to further describe the strength of the relationship between vibratory and DPOAE *Q*_10dB_ values. Paired or unpaired *t* tests were used to compare characteristics of vibratory and DPOAE responses. For all *F* and *t* statistics, degrees of freedom are provided in parentheses. Unless noted otherwise, reported values are the mean ± 1 standard error (SE) and only data with magnitudes exceeding the measurement noise floor are analyzed or plotted. All reported displacement magnitudes are the root-mean-square values.

## Results

### The Tuning Sharpness of Cochlear Vibrations and DPOAE Ratio Functions is Highly Similar

**Fig. 2:**
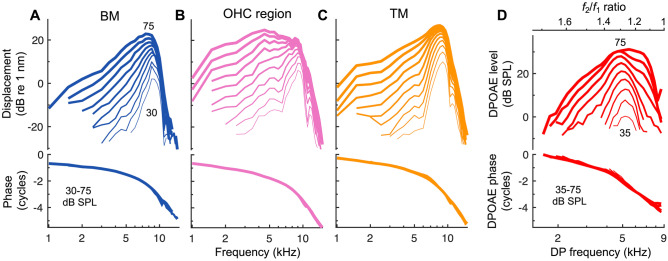
The tuning sharpness of cochlear vibrations and DPOAE ratio functions is level dependent. **A**–**C** Displacement amplitudes (top) and phases (bottom) of the BM (**A**), OHC region (**B**), and TM (**C**) in response to single tones for an individual mouse. Stimuli were swept in frequency (1–15 kHz in 0.5 kHz steps) and level (30–75 dB SPL in 5 dB steps). Responses for higher levels are indicated by increasing line thickness, with 30 and 75 dB SPL responses indicated numerically in (**A**). Phases were referenced to the phase of the stimulus pressure in the ear canal. All responses exhibit compressive, nonlinear growth near the CF and broader tuning with increasing stimulus level. **D** Amplitude and phase of the 2*f*_1_ – *f*_2_ DPOAE measured in the ear canal of the same mouse with *f*_2_ fixed at 9 kHz and *f*_1_ varied. The *f*_2_ and *f*_1_ tones were presented at the same level, from 30 to 75 dB SPL in 5 dB steps, though DPOAEs were typically only measurable above the noise floor for stimulus levels of 35 dB SPL or higher. Phases were referenced to 2*φ*_1ec – _*φ*_2ec_, where *φ*_1ec_ and *φ*_2ec_ were the phases of the *f*_1_ and *f*_2_ stimuli in the ear canal. In all panels, only responses with magnitudes meeting the signal-to-noise criterion are shown (see Methods)

We used OCT to measure sound-evoked vibrations from within the mouse cochlea so that the tuning of the vibratory responses could be compared with the tuning of DPOAE ratio functions from the same ear. Representative displacement responses to single tones are shown for an apical cochlear region tuned to a CF of 9 kHz in Fig. 2A–C. At low stimulus levels, displacements of the BM, OHC region, and TM were all sharply tuned to the CF, while at higher stimulus levels, the responses became more broadly tuned and peaked at slightly lower frequencies. This broadening of tuning with increasing level is due to the inherent nonlinearity and frequency selectivity in the OHC-mediated amplification process, which causes responses near the CF to grow compressively and saturate with increasing stimulus level, and responses at lower frequencies to grow more linearly. Also shown are the displacement phases, which increasingly lag with frequency, reflecting the delays associated with traveling wave propagation.

DPOAE ratio functions obtained from the same ear were also sharply tuned, exhibiting a pronounced bandpass shape (Fig. 2D). With *f*_2_ fixed at 9 kHz and *f*_1_ varied, DPOAE amplitudes reliably peaked at an *f*_2_/*f*_1_ ratio near ~ 1.3 (i.e., when *f*_1_ ≈ 7 kHz and 2*f*_1_ – *f*_2_ ≈ 5 kHz) and rapidly decreased at smaller or larger ratios. While the *f*_2_/*f*_1_ ratio producing the largest DPOAE varied little across stimulus level, DPOAE ratio functions became more broadly tuned as the stimulus level was increased. DPOAE phases also accumulated multiple cycles of lag with increasing DP frequency (i.e., decreasing *f*_2_/*f*_1_ ratio), though the total phase lag was ~ 2–2.5 times that observed for the vibratory responses to single tones over this same frequency range. As the interpretation of DPOAE phases measured using the fixed-*f*_2_, swept-*f*_1_ stimulus paradigm remains uncertain [47], we focus our analyses primarily on DPOAE amplitudes.

**Fig. 3:**
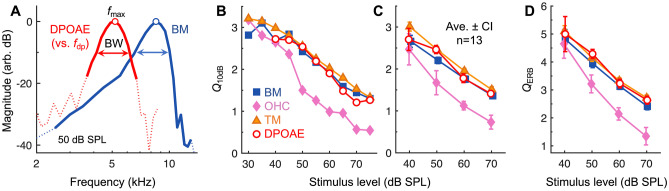
The tuning sharpness of cochlear vibrations and DPOAE ratio functions is highly similar. **A** Single-tone BM responses and DPOAE ratio function amplitudes for stimuli presented at 50 dB SPL. Data are from Fig. 2A, D with DPOAEs plotted vs the DP frequency (*f*_dp_) and both responses normalized to their maximum. Tuning sharpness was quantified by dividing the frequency of the response peak (*f*_max_) by the bandwidth (BW) 10 dB down, yielding the *Q*_10dB_. Data not meeting the signal-to-noise criterion are shown with dotted lines, illustrating that this criterion was strict enough to yield *Q*_10dB_ values that weren’t strongly influenced by noise. **B** *Q*_10dB_ vs. stimulus level for the cochlear vibrations and DPOAE ratio functions shown in Fig. 2. The tuning of the DPOAE ratio functions was similar to BM and TM tuning over a wide range of stimulus levels. OHC region tuning was often broader, particularly at high stimulus levels. **C** Average *Q*_10dB_ (± 95% CI) for cochlear vibrations and DPOAE ratio functions obtained with 40–70 dB SPL stimuli. Averages are from 13 mice in which both the CF and *f*_2_ were 9 kHz. For 40 dB SPL stimuli, DPOAE tuning could only be quantified in seven mice. **D** Average tuning sharpness (± 95% CI) for cochlear vibrations and DPOAE ratio functions expressed in *Q*_ERB_, demonstrating that the correspondence holds for different tuning metrics

To explicitly compare the tuning sharpness of cochlear vibrations and DPOAE ratio functions, we divided the frequency of maximum response for a given stimulus level by the bandwidth 10 dB below to calculate the *Q*_10dB_ value (Fig. 3A). *Q*_10dB_ values for DPOAE ratio functions were remarkably similar to those of BM and TM vibrations across a wide range of stimulus levels, decreasing by a factor of ~ 2 as stimulus levels increased from 40 to 75 dB SPL (Fig. 3B). *Q*_10dB_ values for OHC region vibrations were comparable at stimulus levels below 40 dB SPL, though they tended to decrease more rapidly with increasing stimulus level. The broader tuning of the OHC region is due to a low-pass characteristic that may reflect membrane filtering of the receptor potential, and thus the OHC’s motile response [25, 48].

While the correspondence between *Q*_10dB_ values for DPOAEs and vibratory responses in individual mice was not always as close as that shown in Fig. 3B, we found a strong relationship in the average data (Fig. 3C). Average *Q*_10dB_ values for DPOAE ratio functions were nearly indistinguishable from those of the BM for all stimulus levels except 50 dB SPL, where they were closer to those of the TM. When considering only data obtained with stimulus levels of 50–70 dB SPL, for which *Q*_10dB_ values could be calculated for all measurements in 13 mice, a repeated-measures ANOVA revealed significant effects of both measurement location/type (*F*(1.75,21.04) = 67.01, *P* < 0.001) and stimulus level (*F*(1.37,16.45) = 674.49, *P* < 0.001), and no significant interaction between measurement location/type and stimulus level (*F*(2.46,29.51) = 1.86, *P* = 0.17). Pairwise comparisons between measurement locations/types confirmed that OHC region responses were significantly more broadly tuned than the other types of responses (*P* < 0.001), and BM *Q*_10dB_ values were slightly but significantly lower than TM *Q*_10dB_ (*P* < 0.001). However, *Q*_10dB_ values for DPOAE ratio functions were not significantly different from those of either the BM (*P* = 0.18) or TM responses (*P* = 0.34).

When including data at all levels (40–70 dB SPL in 10 dB steps), DPOAE *Q*_10dB_ values were positively and significantly correlated with BM *Q*_10dB_ (*R*^2^ = 0.83, *P* < 0.001) and TM *Q*_10dB_ (*R*^2^ = 0.81, *P* < 0.001). However, DPOAE and vibratory *Q*_10dB_ values were not significantly correlated when considering the data for any one stimulus level. A weak or absent correlation is not unexpected given that all mice had normal cochlear sensitivity, such that the variance in vibratory or DPOAE responses should be low and perhaps largely attributed to measurement noise. However, we note that the morphology of the DPOAE responses was also somewhat more variable across mice when compared to the vibratory responses, with diverse and nonmonotonic (albeit small) shifts in the optimal *f*_2_/*f*_1_ ratio and DPOAE frequency with changes in stimulus level, which impacted the DPOAE *Q*_10dB_ values. This variable morphology was likely of physiological or acoustic origin, as it was observed even when DPOAE magnitudes were significantly above the measurement noise floor. On average, the variance in DPOAE *Q*_10dB_ values for a given stimulus level was twice that observed in the vibratory responses. Nevertheless, the absolute percentage difference between DPOAE and BM or TM *Q*_10dB_ values obtained in individual ears was at most 32.2%, and only 10.1% on average.

To verify that the similarity in tuning sharpness did not depend on the specific metric used, we also calculated the equivalent rectangular bandwidth (ERB) of each curve. Dividing the frequency of maximum response by the ERB yielded the *Q*_ERB_, which revealed essentially the same close relationship between the tuning of DPOAE ratio functions and the tuning of BM and TM vibrations (Fig. 3D). Average DPOAE *Q*_ERB_ tended to be more similar to TM *Q*_ERB_, rather than BM *Q*_ERB_, although any differences were small. When including data for all stimulus levels, absolute percentage differences between DPOAE and BM or TM *Q*_ERB_ values were 9% on average.

### Tuning of DPOAE Ratio Functions and Cochlear Vibrations is Similar at Higher CFs and *f*_2_ Frequencies

**Fig. 4:**
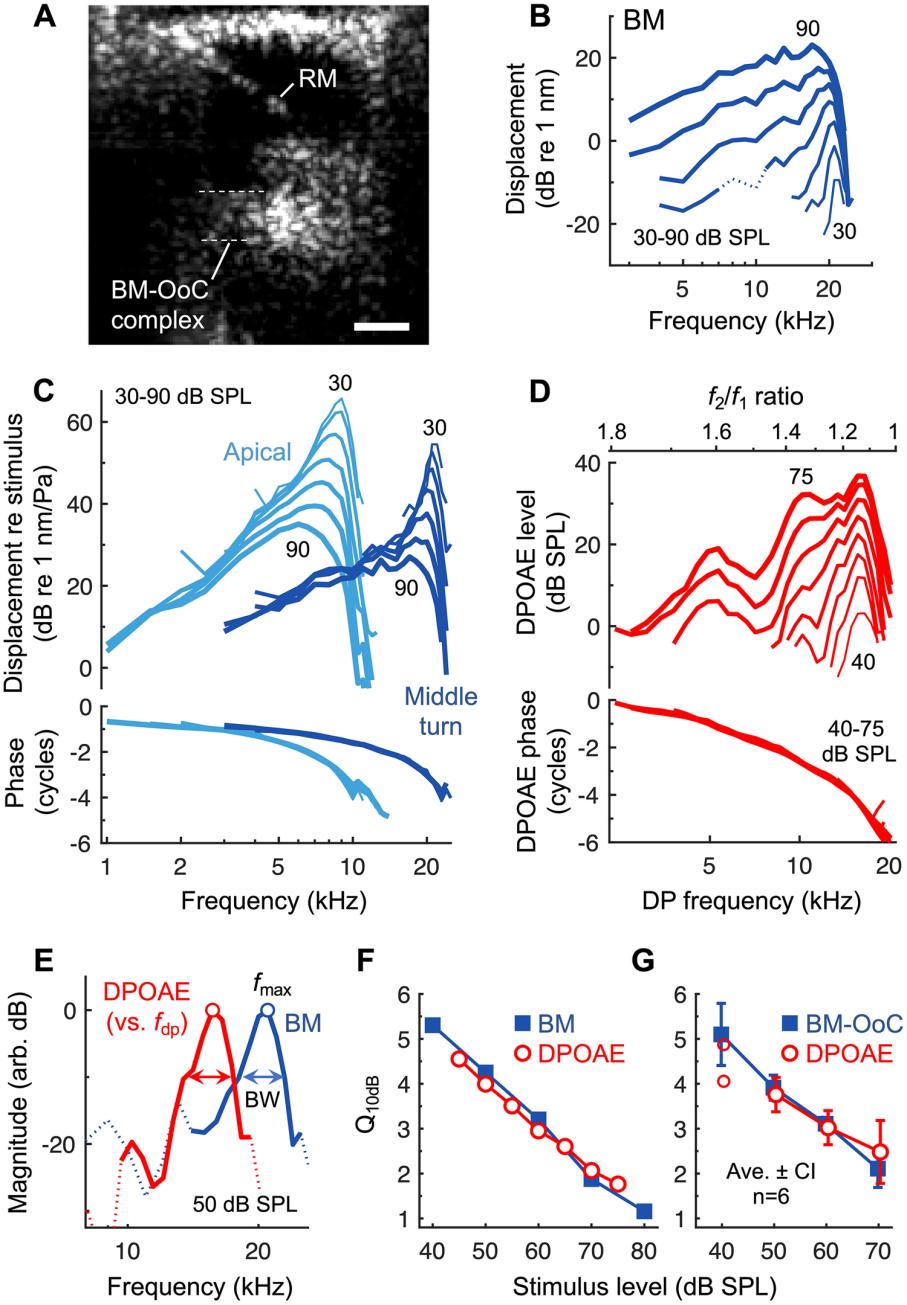
Correspondence between tuning sharpness of cochlear vibrations and DPOAE ratio functions is observed for higher-frequency regions. **A** OCT image of a middle-turn region tuned to 21 kHz. The approximate location of this region within the cochlea is indicated in Fig. 1A. RM is discernible, though the organ of Corti (OoC) is poorly resolved. Measurements were attempted from either the bottom or top of the BM-OoC complex, which is roughly outlined by dotted lines. Scale bar = 100 μm. **B** Displacement responses from the bottom of the BM-OoC complex, presumably near the BM, in an individual mouse. Responses are shown for single tones presented from 30 to 90 dB SPL in 10 dB steps. Responses at the lowest and highest stimulus levels are numerically indicated. Dotted portion of the 60 dB SPL response curve indicates frequencies where data did not meet the signal-to-noise criterion. **C** BM displacements from (**B**) compared with those obtained from the 9 kHz region in the same mouse. Displacements were normalized to the evoking stimulus pressure to highlight the similar degree of nonlinearity near the CF for both apical and middle-turn measurements. Middle-turn displacement phases are consistent with traveling wave propagation to a site closer to the stapes. **D** DPOAE ratio functions from the same mouse with *f*_2_ fixed at the CF (21 kHz) and *f*_1_ varied. Stimuli were varied from 40 to 75 dB SPL in 5 dB steps with the *f*_1_ and *f*_2_ tones presented at the same level. **E** Middle-turn BM responses and DPOAE ratio function amplitudes for stimuli presented at 50 dB SPL. Data are from (**B**) to (**D**) with DPOAEs plotted vs. the DP frequency (*f*_dp_) and both responses normalized to their maximum (circles). Data not meeting the signal-to-noise criterion are indicated by dotted lines. **F** *Q*_10dB_ vs. stimulus level for the middle-turn BM displacements and DPOAE ratio functions shown in (**B**) and (**D**), demonstrating quantitatively similar tuning across stimulus level. **G** Average vibratory and DPOAE *Q*_10dB_ for six mice in which middle-turn measurements were obtained (CF and *f*_2_ = 20–23 kHz). For 40 dB SPL stimuli, the average and 95% CI are not shown for the DPOAE *Q*_10dB_, which could only be quantified in two mice (individual data shown with open symbols)

To test whether the agreement in tuning sharpness extends to other CF regions and *f*_2_ frequencies, we measured vibrations from more basal, middle-turn locations with CFs of 20–23 kHz (average CF = 21.3 kHz) (Fig. 4). Due to the low reflectivity of the structures in the middle turn, the image quality and vibratory signal-to-noise ratio were poorer compared to the apical measurements. However, it was still possible to measure sensitive and sharply tuned vibrations from a region that presumably comprised the BM and organ of Corti (Fig. 4A, B). Vibration measurements were obtained from reflective points at approximately the bottom (*n* = 4) or top (*n* = 2) of this region, likely near the BM or reticular lamina/TM, respectively.

To provide assurance of this, BM responses from the 9 and 21 kHz regions in the same mouse are compared in Fig. 4C, with displacements normalized to the evoking sound pressure to highlight the nonlinearity in the responses. While displacements from the middle turn were smaller than those in the apex, they exhibited a similar amount of nonlinear, compressive growth near the CF. This is demonstrated by the decrease in the normalized response magnitude with increasing stimulus level. For quantitative purposes, we took the difference between the normalized CF responses at 30 and 90 dB SPL as the amount of nonlinear “gain”. Middle-turn gains were 40.5 ± 2.6 dB (*n* = 6), which were comparable to apical BM gains (41.1 ± 0.7 dB, *n* = 13; not significantly different by *t*-test,* t*(17) = 0.28, *P* = 0.78). Gains for the two middle-turn measurements made near the top of the organ of Corti (42.6 and 50.3 dB) were among the three highest observed. This is consistent with the greater gain in TM vs. BM responses found in the apex (TM gain = 44.4 ± 0.5 dB; statistically higher than BM gain by paired *t*-test, *t*(12) = 7.87, *P* < 0.001). Middle-turn displacements also became more broadly tuned and peaked at slightly lower frequencies with increasing stimulus level, though they were always more sharply tuned than the apical responses. Average vibratory *Q*_10dB_ values were ~ 1.5–1.8 times greater in the 20–23 kHz region than in the 9 kHz region, with greater differences at lower stimulus levels. A two-way mixed ANOVA revealed a statistically significant main effect of measurement location (apical vs. middle turn) on *Q*_10dB_ (*F*(1,17) = 321.28, *P* < 0.001) and an interaction between stimulus level and measurement location (*F*(1.74,29.64) = 43.59, *P* < 0.001).

DPOAE ratio functions obtained with *f*_2_ fixed near the CF of the middle-turn measurement site also resembled those obtained with an *f*_2_ of 9 kHz and exhibited a bandpass shape (Fig. 4D; *f*_2_ = 21 kHz). However, they peaked at smaller *f*_2_/*f*_1_ ratios, with an average optimal ratio of 1.14 ± 0.006 (*n* = 6, with optimal ratios first averaged across stimulus level for each mouse). Optimal *f*_2_/*f*_1_ ratios were significantly smaller than those observed for an *f*_2_ of 9 kHz (1.29 ± 0.003, *n* = 13; *t*-test: *t*(17) = 24.03, *P* < 0.001). Ratio functions for *f*_2_ frequencies of 20–23 kHz or 9 kHz therefore peaked when the DPOAE frequency was ~ 0.4 or ~ 0.9 octaves below *f*_2_, respectively. DPOAE ratio functions obtained with high *f*_2_ frequencies also often contained multiple smaller peaks, as reported previously for measurements in gerbil and guinea pig [15, 49, 50]. Notwithstanding this additional complexity, *Q*_10dB_ values could be computed from these measurements that were quantitatively similar to those of the middle-turn displacements (Fig. 4E–G).

For *Q*_10dB_ values derived from middle-turn measurements obtained with stimulus levels of 50–70 dB SPL, a repeated-measures ANOVA (with stimulus level and measurement type as within-subjects factors) revealed no significant main effect of measurement type (*F*(1,5) = 0.11, *P* = 0.75), a significant main effect of stimulus level (*F*(2,10) = 50.35, *P* < 0.001), and a marginally significant interaction between stimulus level and measurement type (*F*(2,10) = 4.88, *P* = 0.03). When also including data collected at 40 dB SPL, for which it was only possible to quantify DPOAE *Q*_10dB_ in two mice, absolute percentage differences between DPOAE and vibratory *Q*_10dB_ values in individual ears were at most 44% and 13.6% on average. Collapsed across stimulus level, DPOAE *Q*_10dB_ values were positively and significantly correlated with vibratory *Q*_10dB_ values (*R*^2^ = 0.75, *P* < 0.001), though correlations were not significant for any given stimulus level. Thus, we observed a quantitative association between DPOAE and vibratory *Q*_10dB_ for high CFs and *f*_2_ frequencies similar to that found when the CF and *f*_2_ were 9 kHz.

### Tuning of DPOAE Ratio Functions is Not Caused by Filtering at the Site of DP Generation

**Fig. 5:**
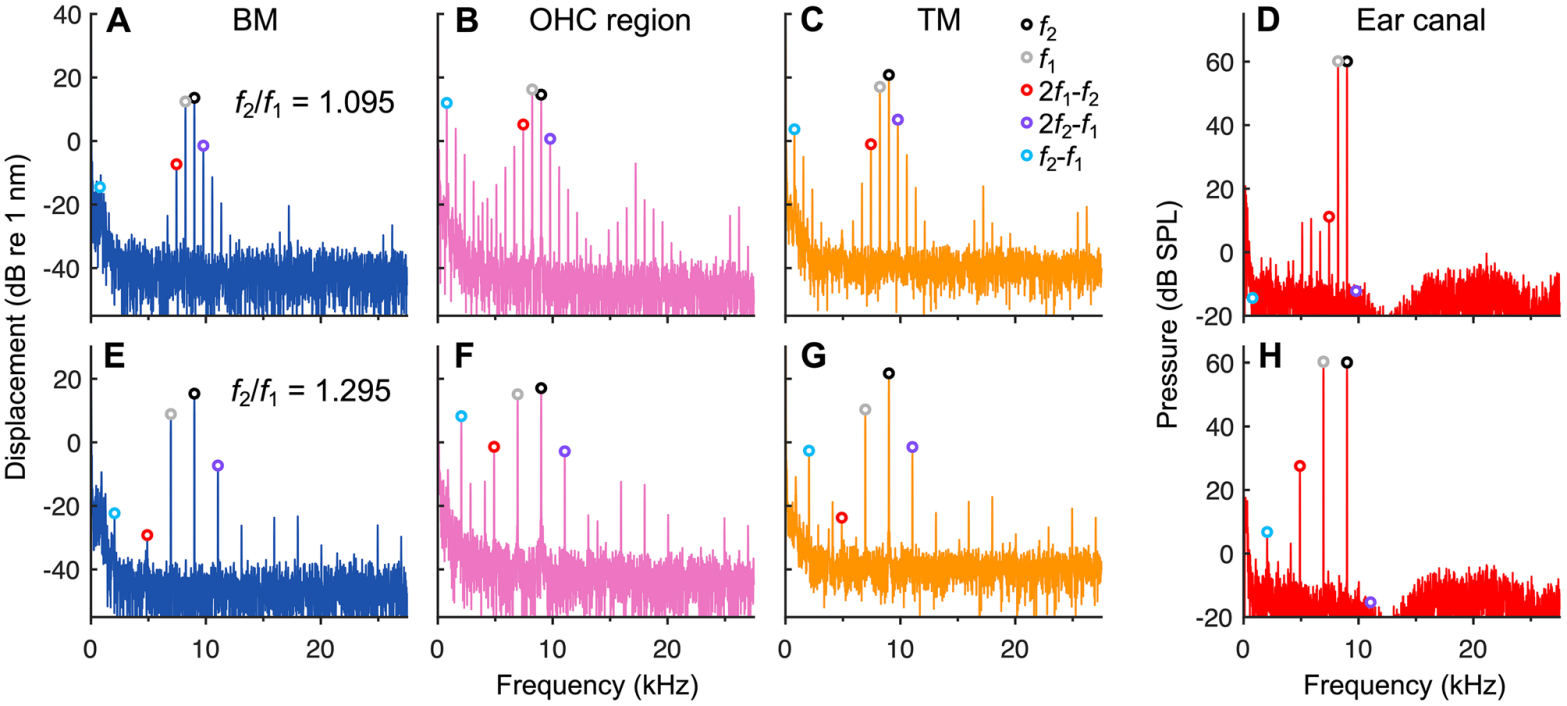
DPs are strongly filtered as they are transmitted within the organ of Corti and to the ear canal. **A**–**C** Displacement spectra for the BM, OHC region, and TM in response to two 60 dB SPL stimulus tones, with *f*_2_ = 9 kHz and *f*_1_ = 8.22 kHz (*f*_2_/*f*_1_ = 1.095) for an individual mouse. Peaks corresponding to responses at *f*_1_, *f*_2_, and several DP frequencies are indicated with circles (legend in **C**). In (**A**), the *f*_2_ – *f*_1_ component is within the noise floor. **D** Ear-canal pressure spectrum from the same mouse. The number and relative magnitudes of DPs measured in the ear canal are very different from those observed in the vibrations. For example, the *f*_2_ – *f*_1_ and 2*f*_2_ – *f*_1_ DPOAEs are within the noise floor despite these components being present in most of the intracochlear recordings. The dip in the microphone spectrum near 12 kHz is the result of correcting for the frequency response of the probe microphone. **E**–**H** Same as (**A**)–(**D**), but with *f*_1_ = 6.95 kHz (*f*_2_/*f*_1_ = 1.295). The 2*f*_1_ – *f*_2_ DP is the largest component in the ear canal but does not dominate the intracochlear DP spectra

To determine whether the tuning of DPOAE ratio functions is due to a mechanism that shapes DPs near where they are generated, we next compared DPOAEs to DPs measured in the vibrations of the BM, OHC region, and TM at the 9 kHz location. Examination of individual two-tone response spectra obtained with *f*_2_ fixed at 9 kHz revealed a complex relationship between the number and amplitude of DP components appearing in the ear canal versus those in the cochlear vibrations (Fig. 5). In general, many more DPs were present in the intracochlear responses than in the ear canal, and DPs at frequencies above *f*_2_ were conspicuously absent from ear-canal spectra. OHC region vibrations exhibited the largest distortions and most broadband distribution of DPs, which were measurable at very low and high frequencies. This confirms recent similar measurements of DPs in gerbil [48, 51] and supports the notion that OHCs are the source of the vibratory DPs. Postmortem measurements verified the physiological origin of the intracochlear DPs and DPOAEs, which were both greatly reduced in magnitude after death and typically not measurable above the noise floor for stimulus levels lower than 70 dB SPL.

While the relative magnitudes of different DP components varied with level, ratio, and measurement location, 2*f*_1_ – *f*_2_ was typically largest in the ear canal but was often not the dominant intracochlear DP component. The dependence of the intracochlear DP magnitudes on measurement location suggests that various but as yet unidentified mechanisms must influence how DPs are transmitted within the organ of Corti. Furthermore, the dissimilarity between ear-canal and intracochlear DP spectra indicates that DPOAEs are strongly filtered by mechanisms which do not shape DPs near where they are generated.

**Fig. 6:**
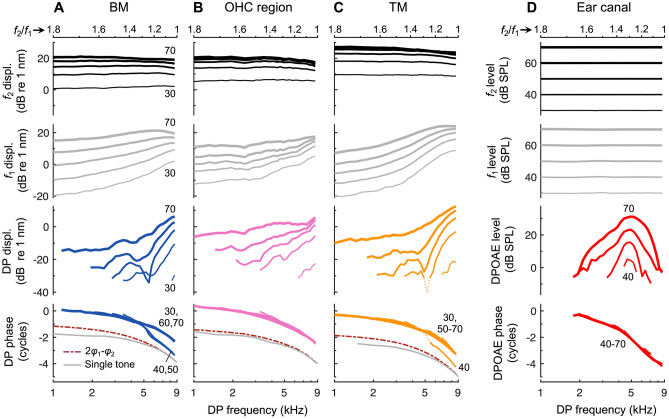
Intracochlear DPs do not exhibit signs of local bandpass filtering. **A**–**C** Displacements of the BM, OHC region, and TM in response to two tones, with *f*_2_ fixed at 9 kHz and *f*_1_ varied, and stimuli presented at the same level (30–70 dB SPL in 10 dB steps; indicated by increasing line thickness and numerically for BM responses). Shown are displacement magnitudes at *f*_2_ (top row), *f*_1_ (second row), and 2*f*_1_ – *f*_2_ (third row), as well as the phase of the 2*f*_1_ – *f*_2_ DP (bottom row). All responses are plotted vs. the DP frequency with the *f*_2_/*f*_1_ ratio axis provided at the top of each column. For comparison are the phases of single-tone responses over the same frequency range, as well as the expected phases of a locally generated DP (i.e., 2*φ*_1 _– *φ*_2_, with *φ*_1_ and *φ*_2_ being the phases of the *f*_1_ and *f*_2_ displacement responses). In (**C**), the dotted portion of the DP response at 40 dB SPL indicates data not meeting the signal-to-noise criterion. **D** As in (**A**)–(**C**) but showing the ear-canal pressure measured in the same mouse. DPOAEs were only measurable at stimulus levels of 40 dB SPL and higher

**Fig. 7:**
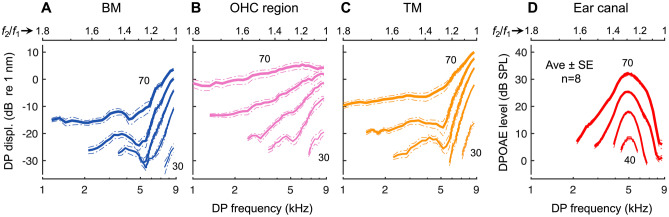
Average intracochlear DP and ear-canal DPOAE ratio functions. **A**–**D** Average 2*f*_1_ – *f*_2_ DP amplitudes measured from the BM (**A**), OHC region (**B**), and TM (**C**), and average DPOAEs (**D**) in eight mice. As in Fig. 6, *f*_2_ was fixed at 9 kHz while *f*_1_ was varied, and stimuli were presented at the same level (30–70 dB SPL in 10 dB steps; indicated by increasing line thickness and numerically for the lowest and highest SPLs). Averages only included responses meeting the signal-to-noise criterion and are shown when such data were available in at least four mice. Dashed-dotted lines indicate ± 1 SE. Average DP phases are not shown, as the stimulus levels for which phase shifts occurred were not the same for all mice. However, the general phase patterns were similar across measurements

As a clear demonstration that DPOAEs undergo filtering not experienced by DPs at their generation site, intracochlear DPs showed no evidence of a bandpass shape as the *f*_2_/*f*_1_ ratio was varied. Intracochlear and ear-canal responses from a representative mouse are shown in Fig. 6, with average data (*n* = 8) shown in Fig. 7. For all intracochlear measurement locations, the 2*f*_1_ – *f*_2_ DP increased as *f*_1_ approached *f*_2_ and was largest at the lowest *f*_2_/*f*_1_ ratios, where DPOAE amplitudes became vanishingly small. The increase in intracochlear DP magnitudes at small *f*_2_/*f*_1_ ratios is consistent with greater nonlinear interactions between the vibrations elicited at the stimulus frequencies, which become more similar in magnitude as *f*_1_ approaches *f*_2_.

DPs measured from the OHC region increased nearly monotonically with decreasing *f*_2_/*f*_1_ ratio, while notches in BM and TM responses were sometimes present at ratios of ~ 1.25–1.3, particularly at lower stimulus levels. Such notches were associated with rapid phase transitions (Fig. 6A, C; bottom row) and may be due to interference between locally generated DPs and DPs that were generated elsewhere and then propagated apically or basally to the measurement site. Indeed, notches occurred near ratios where DPOAEs were largest, suggesting that they may be attributed to the increased strength of a reverse propagating component. Similar notches have been observed in DP ratio functions measured in BM vibrations from gerbil and chinchilla [46, 52].

For OHC region DPs, the lack of prominent notches indicates that these responses were more strongly dominated by a single component – presumably the locally generated DP. DP phases were consistent with the output of a local nonlinearity (dashed-dotted lines in Fig. 6A–C), for which the DP phase takes the form 2*φ*_1_ – *φ*_2_ + *c*, where *φ*_1_ and *φ*_2_ are the phases of inputs at *f*_1_ and *f*_2_, respectively (here taken to be the response phases at *f*_1_ and *f*_2_), and *c* is either 0 or 180 degrees. However, DP phases also grossly resembled the phases of responses evoked by single tones (solid gray lines in Fig. 6A–C), which would be consistent with the responses being dominated by waves traveling through the measurement site at the DP frequency. Thus, it is difficult to disentangle local vs. propagating DP components based on the phase alone.

### Tuning of DPOAE Ratio Functions Is Not Primarily Due to Intracochlear Suppression

In addition to demonstrating the absence of any mechanical resonance that could account for the tuning of DPOAE ratio functions, the intracochlear measurements suggest that the bandpass shape does not arise primarily from nonlinear suppression. When the stimulus tones were presented at the same level, DPOAE amplitudes declined at small *f*_2_/*f*_1_ ratios despite there being little suppression of the *f*_2_ response as *f*_1_ became closer in frequency, and, as noted above, DPs only increased at the smallest *f*_2_/*f*_1_ ratios.

**Fig. 8:**
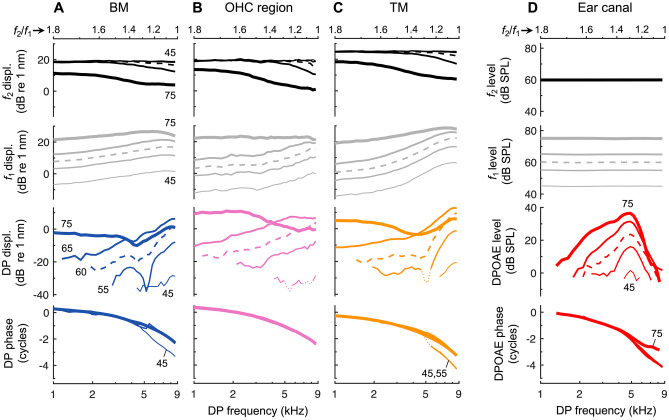
DPOAE ratio functions are not primarily tuned by intracochlear suppression. **A**–**C** Displacements of the BM, OHC region, and TM in response to two tones with the *f*_2_ tone fixed at 9 kHz and presented at 60 dB SPL, and the *f*_1_ tone varied in both frequency and level (45–75 dB SPL in 5 dB steps; data for *f*_1_ levels of 50 and 70 dB SPL not plotted, for clarity). Shown are displacement magnitudes at *f*_2_ (top row), *f*_1_ (second row), and 2*f*_1_ – *f*_2_ (third row), as well as the phase of the 2*f*_1_ – *f*_2_ DP (bottom row) for *f*_1_ tone levels indicated by increasing line thickness and numerically for BM responses. As the level of the *f*_1_ tone increased, the *f*_2_ response was suppressed and DPs could be slightly reduced in magnitude, particularly as *f*_2_/*f*_1_ approached 1. Data are from the same mouse whose responses to equal-level stimuli are shown in Fig. 6. Responses obtained using equal-level stimuli are shown with dashed lines to facilitate comparison. In (**B**) and (**C**), dotted portions of the DP responses indicate data not meeting the signal-to-noise criterion. **D** As in (**A**)–(**C**) but showing the ear-canal pressure. Regardless of the level of the *f*_1_ tone, the decline in DPOAE amplitude at small *f*_2_/*f*_1_ ratios did not resemble that observed for the intracochlear DPs

**Fig. 9:**
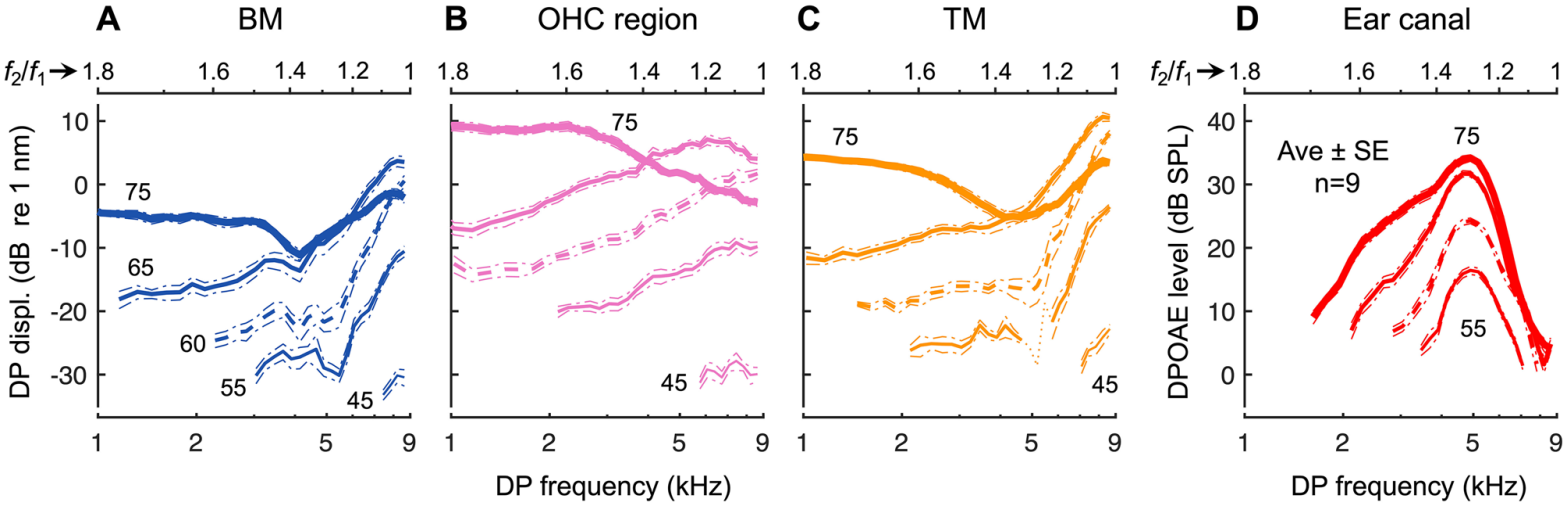
Average intracochlear DP and ear-canal DPOAE ratio functions obtained with unequal vs. equal stimulus levels. **A**–**C** Average 2*f*_1_ – *f*_2_ DP amplitudes measured from the BM (**A**), OHC region (**B**), and TM (**C**), and average DPOAEs (**D**) in nine mice for the paradigm in which the *f*_2_ tone was fixed at 9 kHz and presented at 60 dB SPL, while the *f*_1_ tone was varied in both frequency and level (45–75 dB SPL in 5 dB steps; data for 50 and 70 dB SPL not shown, for clarity). The level of the *f*_1_ tone is indicated by increasing line thickness and numerically in (**A**) (curves for the lowest and highest levels are also labeled in **B**–**D**). Dashed lines indicate data obtained with equal-level stimuli. In (**C**), the dotted portion of the curve for the lowest *f*_1_ level indicates where data from fewer than five mice met the signal-to-noise criterion. Dashed-dotted lines indicate ± 1 SE

Nevertheless, to further explore the potential influence of suppression, we also measured intracochlear DP and DPOAE ratio functions with the level of the *f*_2_ tone fixed at 60 dB SPL and the level of the *f*_1_ tone varied from 45 to 75 dB SPL. Figure 8 shows data obtained with this paradigm from the mouse whose individual responses to equal-level stimuli were plotted in Fig. 6, with average data (*n* = 9) shown in Fig. 9. Presenting the *f*_1_ tone at 60 dB SPL or higher could suppress the *f*_2_ response, with suppression being most prominent when *f*_1_ was close in frequency to *f*_2_. However, even for the highest* f*_1_ stimulus level, DPs measured on the BM still tended to increase at small *f*_2_/*f*_1_ ratios, and TM DPs decreased only slightly under these stimulus conditions. While OHC region DP amplitudes were more clearly reduced with decreasing *f*_2_/*f*_1_ ratio when the level of the *f*_1_ tone was at least 10 dB higher than that of *f*_2_, they started to decline at ratios larger than 1.3 and declined more slowly than DPOAE amplitudes as the *f*_2_/*f*_1_ ratio was decreased. Intracochlear suppression therefore does not appear to play a dominant role in the tuning of the DPOAE ratio functions, which retained their sharp, bandpass characteristic regardless of the amount of suppression observed.

This is not to say that DPOAE ratio functions were uninfluenced by suppression, however. For *f*_2_/*f*_1_ ratios smaller than 1.5, DPOAE amplitudes saturated and decreased as the level of the *f*_1_ tone was raised above ~ 70 dB SPL, likely reflecting the saturation and decline observed in the intracochlear DP magnitudes. DPOAEs obtained with the *f*_1_ tone presented at higher levels therefore tended to decline in amplitude more rapidly as *f*_1_ approached *f*_2_. Since the slope of the ratio functions at large *f*_2_/*f*_1_ ratios simultaneously became less steep, *Q*_10dB_ values underwent small and sometimes nonmonotonic changes as the *f*_1_ stimulus level was increased above 60 dB SPL.

While DPOAE phases were generally similar when obtained with equal- or unequal-level stimuli, presenting the *f*_2_ tone at 60 dB SPL while increasing the level of the *f*_1_ tone produced consistent and large phase leads at small *f*_2_/*f*_1_ ratios. Total phase shifts observed between levels of 60 and 75 dB SPL were maximal near an *f*_2_/*f*_1_ ratio of ~ 1.15 and could approach 1 cycle. These shifts did not appear closely related to changes in the phases of the intracochlear DPs. Complex, level-dependent phase shifts were observed in DPs measured from the BM and TM at ratios where amplitude notches occurred (near an *f*_2_/*f*_1_ of ~ 1.3), while OHC region DPs exhibited consistent, small phase lags (< 0.15 cycles) as the level of the *f*_1_ stimulus exceeded 65 dB SPL. These lags presumably resulted from small phase leads observed in the response at *f*_2_, since the DP phase is expected to follow 2*φ*_1 _– *φ*_2_. Similar phase leads have been previously observed in studies of intracochlear suppression [53–55].

### Wave Interference Produces Bandpass DPOAE Ratio Functions

**Fig. 10:**
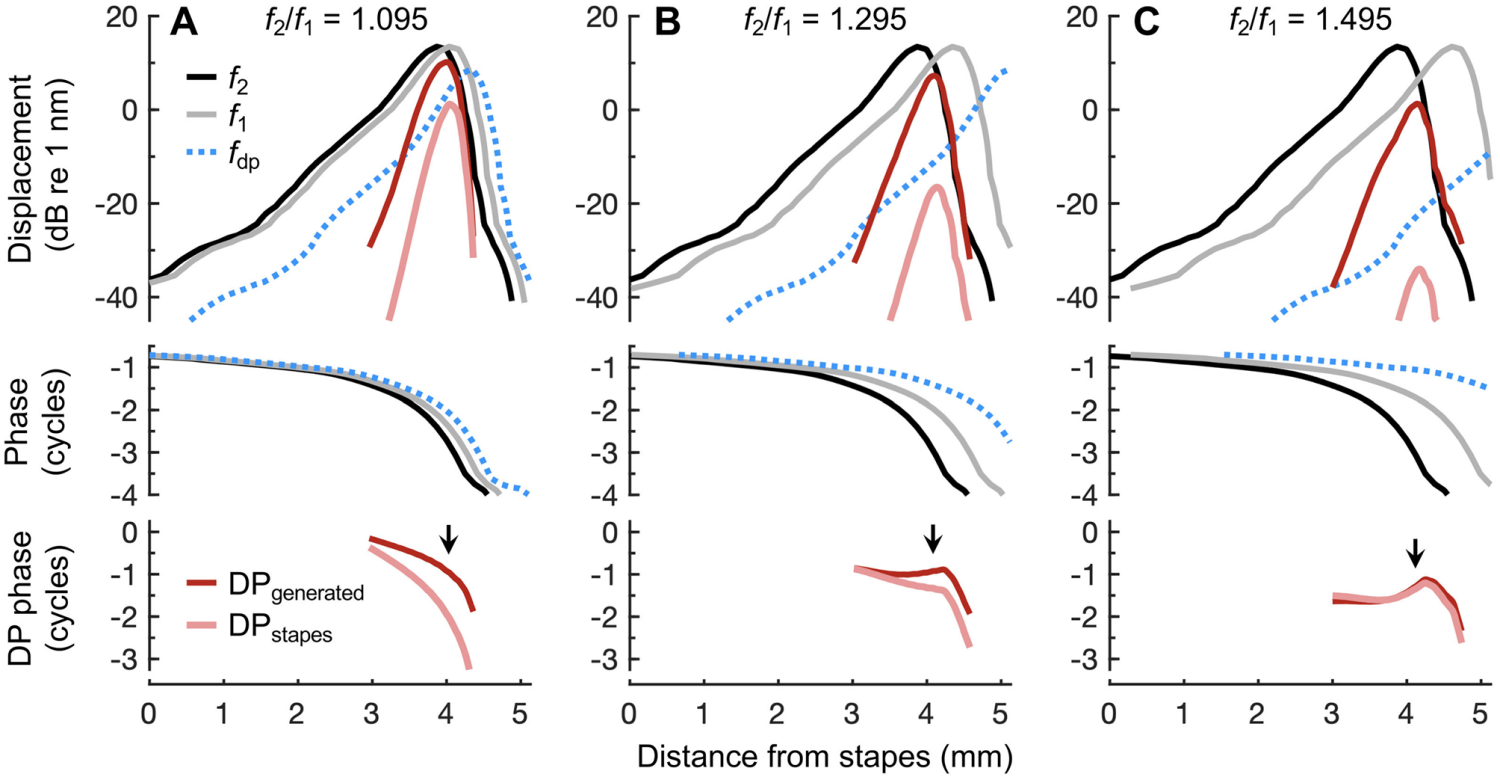
Spatial variation in DP phase predicts destructive interference at small *f*_2_/*f*_1_ ratios. **A**–**C** Estimated BM displacement magnitudes and phases at *f*_1_ and *f*_2_ as a function of cochlear location for *f*_2_/*f*_1_ ratios of 1.095 (**A**), 1.295 (**B**) and 1.495 (**C**), with stimuli presented at 50 dB SPL. At each location, displacements at *f*_1_ and *f*_2_ were used as the inputs to a Boltzmann function, and the response at 2*f*_1_ – *f*_2_ in the low-pass filtered output of the function was taken as locally generated DP (DP_generated_; dark red lines). The DP arriving at the stapes from each location (DP_stapes_; pink lines) was estimated after assuming reverse travel via a wave at the DP frequency (*f*_dp_; dotted blue lines). At small ratios, both the local and propagated DPs have phases that vary rapidly as a function of the generation location, resulting in strong wave interference. At ratios closer to or higher than the experimentally observed optimal ratio, the phases vary more slowly with generation location (arrows indicate phase behavior near the location of maximum DP generation). DP magnitudes are arbitrarily scaled, as the output of the Boltzmann function was normalized to 1. Only DP responses with magnitudes within 40 dB of the maximum DP magnitude are shown. Due to the lower DP frequency in (**B**) and (**C**), the wave at *f*_dp_ peaks abruptly and unrealistically at the apical end of the cochlea. However, this portion of wave falls outside of the DP generation region and is irrelevant to the model’s output

The weak correspondence between intracochlear and ear-canal DPs indicates that the tuning of DPOAE ratio functions is not primarily due to local filtering or suppression of DPs near where they are generated. To demonstrate that this filtering occurs instead through a more global, wave interference phenomenon, we used a simple model to estimate the DPOAEs that would be generated as the *f*_2_/*f*_1_ ratio is varied. First, we approximated the BM’s spatial response to a given pair of *f*_1_ and *f*_2_ tones, with *f*_2_ fixed at 9 kHz, using BM frequency responses from the 9 kHz region (Fig. 10; see Methods). To generate DPs at each cochlear location, the magnitudes and phases of displacements elicited by the *f*_1_ and *f*_2_ tones were used as those of sinusoidal inputs to a Boltzmann function (Eq. (1)), which approximated the nonlinear relationship between OHC stereociliary displacement and transduction current. Since OHC motility is driven by the transmembrane potential, which is subject to electrical filtering by the OHC membrane, we then low-pass filtered the function’s output before determining the magnitude and phase of the resulting 2*f*_1_ – *f*_2_ DP at each location (DP_generated_; i.e., the DP in the OHC’s electromotile output, before it is transmitted to the surrounding structures). Assuming that DPs propagate to the stapes via a wave on the BM, we estimated the magnitude and phase of the DP reaching the stapes from each location (DP_stapes_) by weighting each generated DP’s magnitude by the local BM displacement response to a low-level tone at the DP frequency, and shifting each DP’s phase by the local BM response phase (blue lines in Fig. 10). To approximate the DPOAE in the ear canal, DPs arriving at the stapes from each cochlear location were vectorially summed.

At small *f*_2_/*f*_1_ ratios, the estimated spatial responses reveal that the phases of the generated DPs are predicted to vary rapidly with cochlear location (Fig. 10A; bottom panel). Thus, the resulting DP waves would tend to cancel and produce a small DPOAE. The spatial phase variation of the DPs is rapid because the phases of the waves at *f*_1_ and *f*_2_ (i.e., *φ*_1_ and *φ*_2_) vary rapidly in the region where DP generation is maximal. The locally generated DP phase inherits these phases in the form of roughly 2*φ*_1 _– *φ*_2_, as mentioned previously. The phase variation is even more rapid after accounting for the reverse travel of each DP to the stapes via a wave at the DP frequency, since the DP frequency is close to *f*_1_. In contrast, for *f*_2_/*f*_1_ ratios near the optimum experimentally observed ratio (Fig. 10B), DP phases are predicted to rotate more slowly over the region where DP generation is strongest. Even though the magnitudes of both the locally generated DPs and the DPs at the stapes are reduced compared to the small *f*_2_/*f*_1_ ratio condition, the DP waves should sum more constructively, thus enhancing the DPOAE amplitude.

At larger *f*_2_/*f*_1_ ratios (Fig. 10C), DPOAEs are predicted to decline in amplitude due to the reduced nonlinear interaction between responses at *f*_1_ and* f*_2_, which generates smaller DPs. DPs arriving at the stapes are expected to be even further attenuated by the BM’s low responsiveness to forces generated at the DP frequency (which is ~ 3 kHz when *f*_2_/*f*_1_ = 1.495). At least at low stimulus levels, spatial variation in the DP phases is also predicted to be greater than for the optimal *f*_2_/*f*_1_ ratio, which would result in greater destructive interference between the propagating DPs. At high stimulus levels, this phase variation and the degree of interference are diminished, owing to a reduction in the spatial phase gradient of the response at *f*_2_.

By examining the model output across *f*_2_/*f*_1_ ratios, we found that while maximum intracochlear DP magnitudes tended to increase monotonically as *f*_1_ approached *f*_2_, DPOAE amplitudes peaked and started to decline when the spatial phase variation of DPs propagating from the region of maximum generation to the stapes exceeded 0.5 cycles, in agreement with a previous model of human DPOAE generation [24]. Decreasing the *f*_2_/*f*_1_ ratio both increased the rate of DP phase variation and broadened the generation region, due to the greater overlap between the responses to *f*_1_ and *f*_2_. These two effects synergized to facilitate increasingly destructive interference at small *f*_2_/*f*_1_ ratios.

**Fig. 11:**
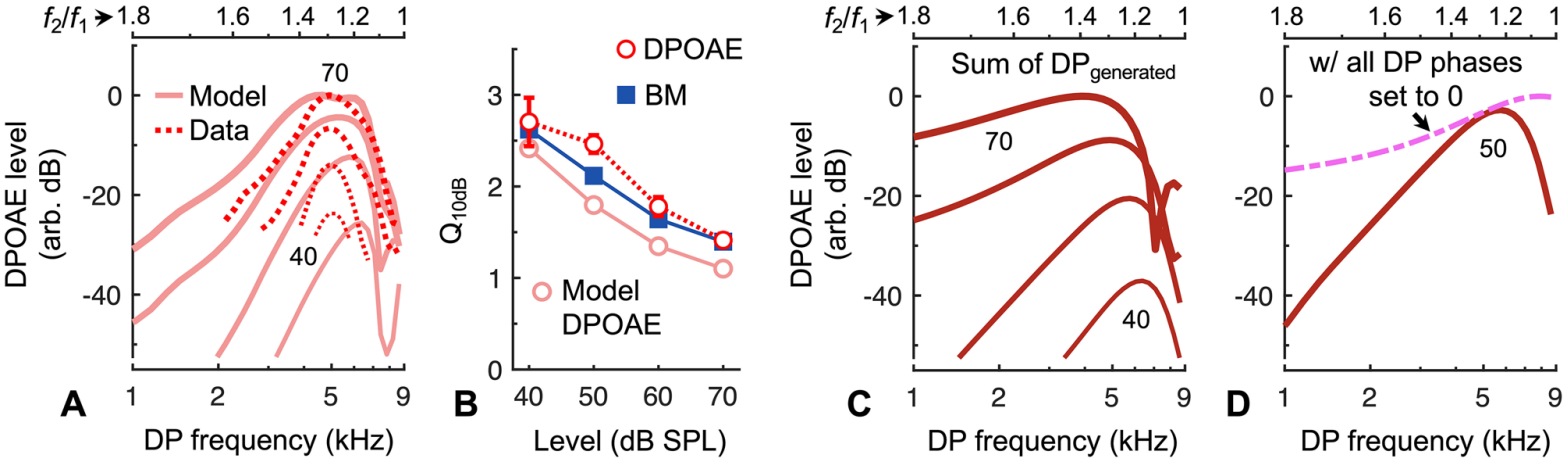
Bandpass tuning of DPOAE ratio functions is caused by wave interference. **A** Modeled DPOAE ratio functions for 40–70 dB SPL stimuli (10 dB steps) exhibit the bandpass tuning observed in the data. Stimulus level is indicated by line thickness and numerically for the lowest and highest levels. For each *f*_2_/*f*_1_ ratio, DPOAEs were estimated by vectorially summing DPs from all locations after assuming reverse propagation to the stapes via a wave on the BM. DPOAE data are averages from Fig. 7D. **B** *Q*_10dB_ values of the modeled DPOAE ratio functions increase with decreasing stimulus level and are similar to, but slightly lower than, *Q*_10dB_ values of the measured DPOAEs (average ± 95% CI from Fig. 3C) and of the average BM displacements used to estimate the spatial responses in the model. **C** Modeled ratio functions still exhibit a bandpass shape when DPOAEs are simply estimated by taking the vector sum of the locally generated DPs (i.e., approximating nearly instantaneous propagation of DPs through the cochlear fluid). The bandpass shape is therefore primarily due to spatial variation in the locally generated DP phase. **D** Comparison of the modeled DPOAE ratio function for 50 dB SPL stimuli from (**C**) with the ratio function obtained when DP phases were all set to 0 prior to vector summation. Without phase variation in the DPs, the resulting DPOAE amplitudes continue to increase with decreasing *f*_2_/*f*_1_ ratio, confirming that bandpass ratio functions arise from wave interference

As shown in Fig. 11A, the modeled DPOAE ratio functions exhibited a bandpass shape that was highly similar to that observed in the data, though the functions were slightly broader and peaked at smaller *f*_2_/*f*_1_ ratios. The optimal ratios for the modeled functions were also level dependent, varying from ~ 1.17 to 1.22. Despite these discrepancies, *Q*_10dB_ values for the modeled DPOAE ratio functions were only ~ 15% lower than those of the average single-tone BM responses used to construct the model, and ~ 21% lower than the measured DPOAE *Q*_10dB_ values (Fig. 11B). This agreement is perhaps surprising, given that the model does not attempt to mimic the actual physics underlying DPOAE generation, and does not explicitly include the effects of amplification or suppression, or the frequency-dependent influence of middle-ear and ear-canal acoustics. Though such adjustments are arbitrary, we could bring the *Q*_10dB_ values of the modeled functions within 5–10% of the measured values by simply eliminating the low-pass filtering step, or by weighting the DPs by BM velocity rather than displacement. Increasing the Boltzmann function’s slope or operating point parameters by a factor of 2 could also produce up to a ~ 10% increase in *Q*_10dB_ when averaged across stimulus level. However, these parameter manipulations had little effect on the optimal ratio and general bandpass shape of the modeled functions.

Modeled ratio functions also retained a bandpass shape regardless of whether DPs were assumed to propagate to the stapes via slow waves on the BM or nearly instantaneously through the fluid (i.e., without weighting the DPs by a BM wave at the DP frequency; Fig. 11C). DPOAE ratio functions produced by simply vectorially summing the locally generated DPs exhibited level-dependent changes in tuning sharpness though were much more broadly tuned, with *Q*_10dB_ values that were ~ 45% lower than the measured values. The propagation route of the DPs may therefore impact the tuning of the ratio functions but is not critical to producing their overall bandpass shape. Instead, the bandpass shape appears to primarily result from spatial variation in the phases of the stimulus-driven waves, which determines the variation in the phases of the generated DPs and, thus, the degree of interference. As expected, setting all DP phases to 0 prior to vector summation resulted in DPOAE amplitudes that increased with decreasing *f*_2_/*f*_1_ ratio, eliminating the bandpass shape (Fig. 11D). Comparison with ratio functions obtained when DP phases were allowed to vary confirmed that destructive interference not only occurs at small *f*_2_/*f*_1_ ratios, but can also be significant at large ratios. Our simple model therefore illustrates that wave interference strongly influences DPOAE amplitudes and is sufficient to account for the bandpass shape in DPOAE ratio functions.

To verify that the above results extended to other cochlear locations, we also used average vibratory responses from the middle turn to estimate spatial responses and DPOAE ratio functions for an *f*_2_ frequency of 20 kHz. When we included low-pass filtering and BM-weighting of the generated DPs, the modeled DPOAE ratio functions for an *f*_2_ of 20 kHz had an optimum *f*_2_/*f*_1_ ratio of 1.1, only slightly smaller than that observed experimentally. *Q*_10dB_ values were 15% lower than those of vibratory responses used in the model and 24% lower than the measured DPOAE *Q*_10dB_, when averaged across stimulus levels of 50–70 dB SPL. The model therefore replicated the decrease in optimal ratio and increase in *Q*_10dB_ at higher *f*_2_ frequencies, with the discrepancy between modeled and measured* Q*_10dB_ values being similar across frequency.

## Discussion

Our measurements demonstrate a striking quantitative agreement between the tuning of DPOAE ratio functions and vibratory responses of the BM and TM to single tones in mice. This agreement was maintained across stimulus level and was observed for two cochlear locations with CFs separated by roughly one octave, when ratio functions were obtained with *f*_2_ fixed at the CF. While variability in the morphology of the ratio functions likely precluded their ability to predict small differences in vibratory tuning among normal-hearing mice, the average DPOAE and vibratory tuning values were found to be highly similar. Thus, at least at the population level, DPOAE ratio functions hold promise as a noninvasive window onto cochlear tuning.

Measurements of intracochlear DPs provide compelling evidence that DPOAE ratio functions in mice are not primarily tuned by mechanisms that locally shape DPs where they are generated — e.g., suppression [16], TM resonance [13], or the form of the DP-generating nonlinearity [17]. This conclusion is supported by findings in other species, for which near-monotonic growth in DP amplitude as* f*_1_ approaches *f*_2_ has been observed in BM vibrations [46, 52], fluid pressure near the BM [56], and, more recently, in vibrations of the reticular lamina [51]. While DPs measured from the TM in the chinchilla apex have also been reported to grow with decreasing *f*_2_/*f*_1_ ratio [57], these measurements were made from a location tuned to the DP frequency, to which DPs had propagated from their more basal generation sites. Here, we show more directly that the TM does not appear to filter the DPs near where they are generated. While we cannot rule out the potential roles of various tuned micromechanical elements or tuning in vibrational modes that are not captured with our approach, the relevance of these features to the generation and propagation of DPs remains uncertain. We also acknowledge that suppression can indeed influence DPOAE ratio functions, through reductions in the magnitudes of the traveling waves at the stimulus and DP frequencies, as well as small phase changes that may alter how the DP waves interfere. However, such effects do not appear critical for producing bandpass ratio functions.

By estimating the spatial profiles of DP sources and the resulting DPOAE amplitudes, we confirmed that the bandpass shape is instead mainly due to a balance between the influence of two factors: (1) the degree of nonlinear interaction between the stimulus-driven waves, which results in the generation of larger DPs with decreasing *f*_2_/*f*_1_ ratio, and (2) wave interference between DPs generated at different locations. More constructive interference between backward-propagating DP waves occurs near the experimentally observed optimal *f*_2_/*f*_1_ ratio, while destructive interference becomes pronounced at smaller *f*_2_/*f*_1_ ratios, if not also at large *f*_2_/*f*_1_ ratios. Similar conclusions regarding the role of wave interference in shaping DPOAEs have been reached previously through numerous modeling efforts [18–21, 24, 58] and experimental studies [59–61]. However, a link between the tuning of DPOAE ratio functions and cochlear vibrations has only recently been explored in a model of human DPOAE generation [24]. This model predicted that the tuning of DPOAE ratio functions should be correlated with, if not quantitatively similar to BM tuning, as confirmed here in mice.

The origin of the precise, quantitative agreement between DPOAE and vibratory tuning requires further examination. As the tuning of the modeled DPOAE ratio functions depended on assumptions and parameters that were independent of the tuning of the model’s responses to the stimulus tones, such an agreement does not appear to be guaranteed. However, at least a correlation between vibratory and DPOAE tuning is anticipated based on the following considerations. First, more sharply tuned responses require smaller *f*_2_/*f*_1_ ratios to elicit nonlinear interactions between the stimulus-driven waves that are sufficient for generating DPs. Second, wave interference between DPs depends on the spatial phase gradients of the waves elicited by *f*_1_ and *f*_2_, which are correlated with tuning sharpness [62, 63]. Specifically, the rate at which *2φ*_1 _*– φ*_2_ varies with cochlear distance plays a critical role, with constructive interference occurring between DP waves when this spatial phase gradient is close to 0, and destructive interference occurring when it significantly departs from this value [20, 21, 24, 58]. Spatial phase gradients can be approximated by the BM phase vs. frequency gradients, which, after being expressed in stimulus periods, increase both with decreasing stimulus level and increasing CF, with greater changes for frequencies near the CF than below the CF. As a result, for more sharply tuned responses, smaller *f*_2_/*f*_1_ ratios are required to achieve gradients in *2φ*_1 _– *φ*_2_ that are close to 0, and the transition from constructive to destructive interference occurs more rapidly with changes in ratio. The ratio functions are therefore tuned to higher DPOAE frequencies and reduced in bandwidth, resulting in higher *Q*_10dB_.

While DPOAE and vibratory tuning co-varied across both stimulus level and frequency, optimal *f*_2_/*f*_1_ ratios decreased with increasing frequency but varied little with changes in stimulus level. For a given CF and *f*_2_ frequency, changes in the bandwidth of the ratio functions therefore primarily drove the correspondence between DPOAE and vibratory tuning across stimulus levels. In humans, optimal *f*_2_/*f*_1_ ratios do decrease at lower stimulus levels [11], though not as dramatically as in modeled ratio functions [24]. The discrepancy between modeled and measured optimal ratios is therefore not unique to the present study and may reflect the influence of mechanisms not included in the models, such as suppression, multiple intracochlear reflections of DP waves, or filtering by the middle ear.

Though we focused on comparing vibratory tuning with the tuning of DPOAE ratio functions obtained with equal-level stimuli, we note that DPOAEs are commonly measured using unequal stimulus levels (e.g., as in [24]). The *f*_1_ tone is typically presented at a higher level than the *f*_2_ tone so as to increase the degree of nonlinear interaction between responses at *f*_1_ and* f*_2_ and maximize the DPOAE amplitude. However, by increasing the spatial extent and strength of DP generation, as well as any suppressive effects of the *f*_1_ tone, the use of unequal levels may affect how DPs interfere and thus change the tuning of the DPOAE ratio functions. Consistent with this, in our measurements where the level of the *f*_2_ tone was fixed at 60 dB SPL, increasing the level of the *f*_1_ tone had complex effects on DPOAE ratio function morphology (Figs. 8 and 9). More comprehensive exploration of the parameter space would be useful for understanding the origins of such effects and for optimizing the correlation between DPOAE and vibratory tuning.

Comparisons of DPOAE and vibratory tuning in other species are necessary to determine if a quantitative agreement between the two is a fundamental characteristic of the mammalian cochlea’s operation. If so, this may serve as an important constraint on future models, possibly clarifying aspects of OAE generation and propagation. Especially interesting would be measurements in animals whose hearing extends to lower frequencies, as apical responses in such species are much more broadly tuned [64–66], and the mechanics involved in DPOAE generation in these regions may be different. In humans and guinea pigs, the tuning sharpness of DPOAE ratio functions decreases two-fold as *f*_2_ is lowered from ~ 10 to 1 kHz [23, 67], suggesting that a correlation between DPOAE and cochlear tuning could extend to apical regions. However, such correlations may be obscured by the fact that ratio functions from other species are often more complex than those reported here, containing multiple strong peaks or sharp notches that are indicative of interference [15, 49, 68]. The origin of these features and how to best avoid or remove them requires further attention.

Whether DPOAE ratio functions can be used to infer cochlear tuning in ears with OHC dysfunction, and presumably broader tuning, is also of key interest. While OHC insult typically leads to reduced DPOAE amplitudes, reported effects on DPOAE ratio functions have been less straightforward. Bandpass tuning is generally preserved in ratio functions from rabbits following noise exposure [69] and from gerbils and mice after treatment with furosemide, an ototoxic drug [50, 70]. Ratio functions from mice with OHC dysfunction have been found to either change little [70] or exhibit complex changes related to the amount of hearing loss, with shifts toward larger optimal *f*_2_/*f*_1_ ratios for moderate losses and de-tuned or low-pass functions (when plotted vs. DP frequency) for more severe losses [71]. For mutant mice in which the TM is detached from the OHCs, ratio functions either retain a bandpass shape or become high-pass [72]. Similarly mixed results have been observed in humans with permanent or temporary hearing loss [73–75].

As direct mechanical measures were not performed in any of the aforementioned studies, conclusions regarding the utility of ratio functions for assessing mechanical tuning in impaired ears are tentative. Still, it is conceivable that diverse patterns of OHC damage produce similarly diverse effects on how DP waves interfere, leading to complex effects on ratio function morphology. Additionally, certain mutations affecting the TM and OHC stereocilia have actually been found to increase the active or passive tuning of cochlear vibrations [76, 77]. Further measurement and modeling of responses in impaired ears are therefore necessary to assess how ratio functions from such ears specifically relate to mechanical tuning. Such work should carefully consider how the spatial extent of DP generation and the DP propagation route may be affected in ears with OHC dysfunction, particularly at the higher stimulus levels that are required to elicit responses in these ears.

At least in normal-hearing mice, our comparisons of intracochlear and ear canal DPs (Fig. 5) support the view that DPs propagate to the stapes via slow, BM-coupled traveling waves. Primary evidence for this was that DP components at frequencies significantly above *f*_2_ (with *f*_2_ = CF) were abundant inside of the cochlea but nearly absent in the ear canal. This is presumably because the BM within the DP generation region cannot support slow, traveling waves at frequencies much higher than the CF. If DPs instead propagated mainly through fast waves in the fluid, one might expect to measure more of the high-frequency components in the ear canal. Assuming that DPs in the motions of the OHC region (or TM) are not directly coupled to the stapes through the fluid, then the transmission of lower-frequency DPs to the ear canal is likely also shaped by BM mechanics. Thus, even if OHCs in basal regions produce 2*f*_1_ – *f*_2_ DPs in response to low-frequency stimuli, the contribution of these components to the total reverse-propagating DP wave will be attenuated by the underlying BM’s weak response to forces at the DP frequency, which will fall far below the local CF. The BM and its fluid interactions are therefore expected to restrict the basal extent of DPs contributing significantly to the DPOAE, as suggested by recent modeling work [38, 78]. Whether alternative modes of DP propagation and/or contributions from basal OHC sources become more influential under certain stimulus conditions or in pathological ears remains to be determined.

## Data Availability

All data used to support the conclusions in this manuscript are available at https://github.com/jbdewey/Dewey2023JARO.
